# Personalia

**Published:** 1913-11-08

**Authors:** 


### Personalia.

Mr. George Dew has resigned his membership of the Manor Mental Hospital Sub-Committee of the London County Council.

Mr. G. W. B. James and Mr. B. F. Beatson have resigned their appointments as fourth and sixth assistant medical officers respectively at the Hanwell Mental Hospital.

Lady Wernher has been elected a patroness of the Seaside Convalescent Hospital, Seaford, Sussex. The committee of management resolved that one of the wards in the hospital be henceforth known as the "Wernher Ward," in recognition of the interest and generosity shown to the institution by the late Sir Julius Wernher.

Sir M. M. Bhownaggree has offered to the London School of Tropical Medicine a medal to be competed for annually. This medal is provided from a fund raised to perpetuate the memory of the late Dr. Cawas Lalcaca, who lost his life in London in April 1909. The medal, which will be awarded at the annual dinner of the School, will be open to competition by qualified medical graduates of any nationality who have attended a full course at the School, who are reported upon as having been diligent throughout this period, and who have obtained the School certificate.

